# Baseline blood pressure does not modify the effect of intravenous thrombolysis in successfully revascularized patients

**DOI:** 10.3389/fneur.2022.984599

**Published:** 2022-09-12

**Authors:** Zhang Xiaoxi, Zhu Xuan, Zhang Lei, Li Zifu, Xing Pengfei, Shen Hongjian, Zhang Yongxin, Hua Weilong, Zhou Yihan, Dai Dongwei, Li Qiang, Zhao Rui, Huang Qinghai, Xu Yi, Lili Song, Craig S. Anderson, Liu Jianmin, Zhang Yongwei, Yang Pengfei

**Affiliations:** ^1^Neurovascular Center, Changhai Hospital, Naval Medical University, Shanghai, China; ^2^Global Brain Health, The George Institute for Global Health, Beijing, China; ^3^The George Institute for Global Health, Faculty of Medicine, University of New South Wales, Sydney, NSW, Australia; ^4^Stroke Program, The George Institute for Global Health, Beijing, China; ^5^Department of Neurology, Royal Prince Alfred Hospital, Sydney Health Partners, Sydney, NSW, Australia

**Keywords:** baseline blood pressure, endovascular thrombectomy, intravenous thrombolysis, acute ischemic stroke, endovascular treatment

## Abstract

**Background:**

Studies indicate a trajectory relationship between baseline blood pressure (BP) and outcome in patients with acute ischemic stroke (AIS) eligible for both intravenous thrombolysis (IVT) with alteplase and endovascular treatment (EVT). We determined whether baseline BP modified the effect of IVT in successfully revascularized AIS patients who participated in the Direct Intra-Arterial Thrombectomy to Revascularize AIS Patients With Large Vessel Occlusion Efficiently in Chinese Tertiary Hospitals (DIECT-MT) trial.

**Methods:**

The association of baseline systolic BP, trichotomized as high (141–185 mmHg), middle (121–140 mmHg), and low (91–120 mmHg), and the outcomes of any intracerebral hemorrhage (ICH), symptomatic ICH (sICH), and mortality and functional outcome on the modified Rankin scale at 90 days were explored. Logistic regression models determined the interaction between clinical outcomes and baseline systolic and diastolic BP, and mean arterial pressure (MAP), at 10 mmHg intervals. Data are reported as odds ratios (OR) and 95% CI.

**Results:**

A *post-hoc* analysis of DIRECT-MT, in 510 of the 656 randomized participants with successful revascularization underwent MT. The overall adjusted common OR of IVT and baseline BP on any ICH, sICH, and 90-day mortality and functional outcome were 0.884 (95%CI 0.613–1.274), 0.643 (95%CI 0.283–1.458), 0.842 (95%CI 0.566–1.252), and 1.286 (95%CI 0.772–2.142), respectively. No significant interaction between baseline blood pressure and intravenous thrombolysis with clinical outcome was observed.

**Conclusions:**

In patients with baseline SBP under 185 mmHg, baseline blood pressure does not alter the risk of hemorrhagic transformation and clinical

outcome in successfully revascularized patients, regardless of intravenous alteplase usage. Future studies are needed to confirm our findings.

**Registration:**

URL: http://www.clinicaltrials.gov, Identifier: NCT03469206.

## Introduction

The functional outcome of patients who have received endovascular thrombectomy (EVT) alone is non-inferior to those who have had EVT after receiving conventional bridging thrombolysis, despite a higher likelihood of successful revascularization with the latter but no clear difference in the risk of symptomatic intracranial hemorrhage (sICH) (1). However, successful EVT is complicated by a number of factors, among which peri-procedural hypertension is common and associated with greater hemorrhagic transformation, poor functional outcome, and death (2–4). Although several studies indicate a U- or J-shaped association of baseline BP and functional outcome after EVT, the baseline BP nadir differed from studies. *Post-hoc* analysis (5) of MR CLEAN trial, for example, found a U-shaped relation between baseline systolic BP and functional outcome, with the nadir at 120 mmHg, while the SITS (6) and ETIS (7) registries showed that systolic BP of 141–150 and 150–160 mmHg, respectively, were associated with the most favorable outcome. *Post-hoc* analysis of the PASS study also showed that low SBP at baseline was associated with an increased risk of in-hospital mortality and complications, and the cutoff for low SBP was 130 mmHg (8). BP-TARGET was the first randomized clinical trial investigating intensive systolic blood pressure targets (100–129 mm Hg) compared with a standard target (130–185 mm Hg) after successful endovascular therapy, finding no significant difference in radiographic intra-parenchymal hemorrhage rate or favorable clinical outcome (9). Moreover, it remains uncertain whether baseline BP influences the effect of IVT on the risk of ICH as well as the long-term functional outcome. We therefore undertake a secondary analysis to determine the association between baseline BP and functional outcome in a cohort of successfully revascularized AIS patients and the interaction between IVT and baseline BP in the DERECT-MT trial.

## Materials and methods

### Design

Details of the study protocol for the direct intra-arterial thrombectomy to revascularize AIS patients with large vessel occlusion Efficiently in Chinese Tertiary Hospitals: a Multicenter Randomized Clinical Trial (DIRECT-MT) are published elsewhere (10). In brief, DIRECT-MT was a multicenter randomized controlled, blinded outcome assessed, clinical trial that investigated the effects of direct vs. conventional prior intravenous alteplase on MT in eligible patients within 4.5 h of the onset of symptoms at 41 hospitals in China from 23 February 2018 to 2 July 2019. The interventions were assessed in a non-inferiority design on a background of standard stroke care delivered according to national guidelines. In this *post-hoc* analysis, included patients were those who had achieved successful revascularization, defined by eTICI scores 2b/2c/3. Approvals were obtained from site ethics committees prior to any data collection and informed consent was obtained from all patients or their legal representatives prior to randomization.

### Assessments

Baseline characteristics included demographic and clinical information, including neurological severity on the NIHSS scale and various time performance parameters in relation to EVT, which were extracted from the DIRECT-MT database. Baseline systolic BP (SBP) and diastolic BP (DBP) were measured at the time of presentation to the emergency department, and SBP was trichotomized into three groups as high (141–185 mmHg), middle (121–140 mmHg), and low (91–120 mmHg). The mean arterial pressure (MAP) was calculated with the following formula: MAP = 2/3^*^DBP + 1/3^*^SBP. A routine automatic measurement of arterial BP was performed. Baseline BP exceeding 185/110 mmHg was a contraindication for IVT, therefore, patients with baseline BP exceeding this interval were excluded from this study.

### Outcome and safety measures

The primary analysis focused on any interaction between baseline BP and the effect of IVT on the primary outcome of this trial, in which the functional outcome was measured by the modified Rankin Scale (mRS) at 90 days. A favorable functional outcome was defined as mRS 0–2. The investigated population was mainly successfully recanalized patients who were extracted from pooled data of DIREC-MT database. Secondary outcomes included favorable functional outcomes, defined as an mRS score of 0–2 at 90 days, and sICH. ICH was classified according to criteria. Symptomatic ICH was defined as a worsening of NIHSS with a score increased for 4 points or more within 24 h attributable to ICH.

### Statistical analysis

Baseline clinical variables are presented with descriptive statistics using frequencies and percentages for categorical variables and medians and interquartile ranges (IQRs) for continuous variables as appropriate. No imputation was performed for missing data since the missing data were minimal. The absolute benefit of intravenous alteplase for different BP values (trichotomized) was computed using the estimated probability of good functional outcome (mRS 0–2) for the intervention and control arm. The interaction between baseline BP and IVT was tested with multivariable ordinal logistic regression analysis with an interaction term. We computed (common) ORs per 10 mmHg SBP increase to assess the relation of SBP with the outcome on the mRS, mortality and ICH; as dichotomized (mRS 0–2 vs. 3–6, logistic regression analysis). Adjusted odd ratios were reported as effect size estimates with their respective 95% CIs. All *P*-values were 2-sided, and conventional levels of significance (α, 0.05) were used for interpretation. All analyses were performed using the SAS software, version 9.4 (SAS Institute).

## Results

### Patients and clinical outcomes

A total of 656 patients were randomized in the DIRECT-MT trial, and 510 were successfully revascularized after MT with or without intravenous thrombolysis. The majority of participates were admitted with a higher admission SBP of 140–185 mmHg (*n* = 314, 61.57%), who were older and more likely to have a hypertension history (Table 1). No significant difference in IVT usage rates of low, middle, and high groups (50.94 vs. 54.55 vs. 51.27%) was observed. The median time from door to intravenous alteplase, time from door to groin puncture and time from onset to revascularization were also not significant.

**Table 1:** Baseline characteristics of successfully revascularized patients.

**Baseline characteristics**	**Low (*n =* 53)**	**Middle (*n =* 143)**	**High (*n =* 314)**	***P*-trend**
Age	62 (52,71)	67 (58,74)	71 (64,78)	<0.001
Male	33 (62.26%)	90 (62.94%)	171 (54.46%)	0.182
Hypertension	22 (41.51%)	66 (46.15%)	222 (70.70%)	<0.001
Diabetes mellitus	10 (18.87%)	26 (18.18%)	59 (18.79%)	0.987
Location of occlusion				0.97
ICA	19 (35.85%)	54 (37.76%)	109 (34.71%)	
M1 segment	29 (54.72%)	77 (53.85%)	174 (55.41%)	
M2 segment	5 (9.43%)	12 (8.39%)	31 (9.87%)	
Cause of stroke				0.971
Cardioembolism	20 (37.74%)	67 (46.85%)	141 (44.90%)	
Intracranial atherosclerosis	2 (3.77%)	10 (6.99%)	30 (9.55%)	
Ipsilateral extracranial ICA occlusion	6 (11.32%)	13 (9.09%)	29 (9.24%)	
Undetermined	25 (47.17%)	53 (37.06%)	114 (36.31%)	
ASPECTS at admission, median (IQR)	9 (7,10)	8 (7,10)	9 (7,10)	0.654
Intravenous Alteplase	27 (50.94%)	78 (54.55%)	161 (51.27%)	0.796
Time from door to IVT, median (IQR)	72 (51,90)	55 (45,77)	56.5 (42,73)	0.166
Time from door to groin puncture, median (IQR)	84 (66,107)	82 (65,101)	85 (70,104)	0.419
Time from onset to revascularization, median (IQR)	285 (205,345)	264 (217,316)	268 (223,315)	0.603
NIHSS at admission, median (IQR)	17 (14,20)	17 (14,21)	17 (13,22)	0.692
NIHSS at 5–7 days or discharge, median (IQR)	5 (1,13)	6 (1,15)	7 (2,16)	0.454
Baseline SBP, median (IQR)	110 (105,117)	131 (123,135)	158 (149,170)	<0.001
Baseline DBP, median (IQR)	70 (61,77)	79 (72,85)	89 (80,99)	<0.001
Any ICH	25 (47.17%)	55 (38.46%)	129 (41.08%)	0.545
sICH	1 (1.89%)	11 (7.69%)	15 (4.78%)	0.220
90d mRS 0-2	25 (47.17%)	62 (43.36%)	120 (38.34%)	0.358
90d mortality	7 (13.21%)	24 (16.78%)	47 (14.97%)	0.799

ASPECTS, Alberta Stroke Program Early CT Score; DBP, diastolic blood pressure; ICH, intracranial hemorrhage; IVT, intravenous thrombolysis/alteplase; SBP, systolic blood pressure.

The occurrence of any ICH was slightly higher in the low BP group with a proportion of 47.17% (25/53), compared with 38.46% (55/143) and 41.08% (129/314) in the middle and high BP groups, however, with no significance (*P* = 0.545). Conversely, the occurrence of symptomatic ICH was lowest in the low BP group with a proportion of 1.89% (1/53). Symptomatic ICH occurred in 7.69% (11/143) and 4.78% (15/314) patients in the middle and high BP groups. A total of 47.17% (25/55) patients of the low BP group achieved functional outcomes at 90 days of follow-up and 13.21% (7/55) suffered death.

### Interaction between intravenous alteplase and baseline SBP

**Figure 1:**
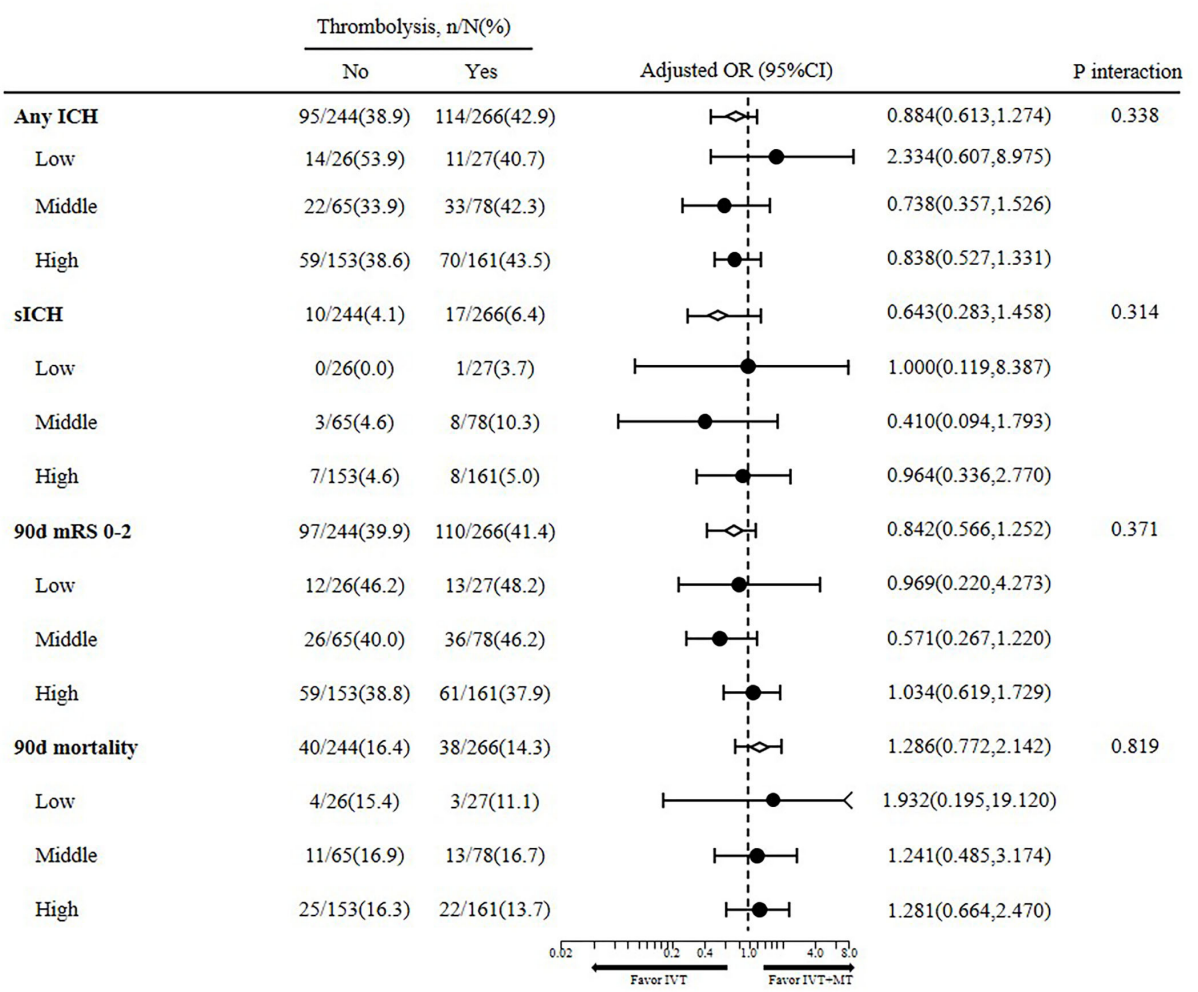
Interaction between baseline blood pressure with intravenous alteplase on clinical outcome.

The key prognostic covariables included baseline NIHSS score; baseline ASPECTS score; history of hypertension, diabetes mellitus, and atrial fibrillation. After adjustment, we found no interaction between baseline SBP and the effect of intravenous alteplase on the functional outcome (*P* = 0.371) (Figure 1). The effect of intravenous alteplase was similar in the low (aOR = 0.969, 95%CI 0.220–4.273), middle (aOR = 0.571, 95%CI 0.267–1.220), and high BP groups (aOR = 1.034 95%CI 0.619–1.729). There was no interaction between baseline SBP and the effect of intravenous alteplase on 90 days mortality (*P* = 0.819). The effect of intravenous alteplase is comparable in three groups (aOR = 1.932, 95%CI 0.195–19.120; aOR = 1.241, 95%CI 0.485–3.174; aOR = 1.281, 95%CI 0.664–2.470).

Furthermore, baseline SBP did not influence the intravenous alteplase effect on the occurrence of sICH (*P* = 0.314) or any ICH (*P* = 0.338). However, lower baseline SBP showed a lower trend for better 90d functional outcome in both thrombolysis (46.2 vs. 40.0 vs. 38.8%) and non-thrombolysis patients (48.2 vs. 46.2 vs. 37.9%), however, without statistical significance (*P* = 0.371).

### Association between baseline BP and clinical outcomes

As shown in Table 2, no interaction was observed between baseline BP and clinical outcomes (*P* > 0.05). With baseline SBP 90–185 mmHg, no relationship was observed between baseline SBP per 10 mmHg and sICH (aOR = 0.982, 95%CI 0.814–1.184, *P* = 0.846) and any ICH (aOR = 0.976, 95%CI 0.897–1.061, *P* = 0.566). DBP and MAP were also not related to sICH and any ICH as shown in Table 2 (*P* > 0.05). Considering the improvement of NIHSS score from admission to day 5–7 or discharge, SBP, DBP, and MAP showed no influence (coefficient = 0.126, 95%CI −0.336 to 0.589, *P* = 0.593; coefficient = 0.107, 95%CI −0.590 to 0.805, *P* = 0.763; coefficient = 0.156, 95%CI −0.518 to 0.831, *P* = 0.650). Functional outcomes of mRS 0 to 2 and mortality at 90 days were also not related to BP, as shown in Table 2.

**Table 2:** The interaction between baseline blood pressure and clinical outcome.

**BP and sICH^a^**	**aOR**	**95% CI**	***p*-value**
SBP per 10 mmHg	0.982	0.814–1.184	0.846
DBP per 10 mmHg	1.029	0.783–1.353	0.836
MAP per 10 mmHg	1.006	0.767–1.318	0.967
**BP and any ICH** ^**a**^	**aOR**	**95% CI**	***p*** **-value**
SBP per 10 mmHg	0.976	0.897–1.061	0.566
DBP per 10 mmHg	0.970	0.854–1.102	0.643
MAP per 10 mmHg	0.964	0.853–1.091	0.564
**BP and** **ΔNIHSS shift**^**a**^	**Coefficient**	**95% CI**	***p*** **-value**
SBP per 10 mmHg	0.126	−0.336,0.589	0.593
DBP per 10 mmHg	0.107	−0.590,0.805	0.763
MAP per 10 mmHg	0.156	−0.518,0.831	0.650
**BP and mRS=0** **~** **2** ^**a**^	**aOR**	**95% CI**	***p*** **-value**
SBP per 10 mmHg	0.976	0.890–1.071	0.613
DBP per 10 mmHg	0.906	0.790–1.041	0.163
MAP per 10 mmHg	0.925	0.810–1.057	0.253
**BP and 90d mortality** ^**a**^	**aOR**	**95% CI**	***p*** **-value**
SBP per 10 mmHg	0.941	0.837–1.059	0.315
DBP per 10 mmHg	0.968	0.805–1.164	0.731
MAP per 10 mmHg	0.935	0.782–1.118	0.464

^a^Adjusted for key prognostic covariates (age; baseline NIHSS score; baseline ASPECTS score; history of hypertension, diabetes mellitus and atrial fibrillation).

## Discussion

This *post-hoc* analysis was driven by uncertainty over whether baseline BP affects the benefit of intravenous alteplase before mechanical thrombectomy, given that the risk of thrombolysis-related intracerebral hemorrhage was relatively high in patients with high blood pressure (3, 4). The main finding of this study was that the baseline BP was not associated with the benefit of IVT in eligible patients (SBP <185 mmHg) for both IVT and EVT. The usage of IVT does not affect the relation between baseline BP and clinical outcome. Namely, IVT may not be used as a predictor of clinical outcomes in successfully revascularized AIS patients.

Successful or failed recanalization status influences the interaction between BP in the acute phase and functional outcome. In historical stroke cohorts, a U- or J-shaped relationship between baseline BP and clinical outcomes were identified. However, most of these studies predated current revascularization strategies, disregarding the recanalization state of the affected arterial territory. In the ETIS registry (6), high SBP 2–24 h after thrombolysis had a linear association with symptomatic hemorrhage and a U-shaped association with mortality and independence with SBP 141–150 mmHg associated with most favorable outcomes, and the nadir was 157 mmHg (95%CI 143–170). Martins et al. (11) performed a multivariate analysis of SBP and DBP in patients according to recanalization status, finding a linear association with functional outcome (SBP: OR = 1.015, *P* = 0.001 DBP: OR = 1.019 *P* = 0.012) in recanalized patients and a J-shaped relationship with non-recanalized patients. In this study, we found no significant relationship between baseline BP and clinical outcome. The reason might be the “plateau effect," literally, in patients with baseline BP <185/100 mmHg, the trajectory effect is not significant. Furthermore, for patients eligible for IVT + EVT, neither baseline BP nor IVT usage affect the risk of poor functional outcome, and cannot be used as a predictor of clinical outcome. Nevertheless, patients with baseline BP exceeding 185/100 mmHg were excluded because of contraindications to intravenous alteplase. We assume that patients with baseline BP exceeding the normal range might suffer a higher risk of symptomatic ICH and poor clinical outcomes because of BP while not using alteplase.

Systolic blood pressure at admission is an important predictive factor for clinical outcome after acute stroke. Higher baseline BP was associated with the better collateral flow after stroke onset, however, did not translate into better clinical outcomes. A meta-analysis recruiting 26 studies indicated that pre-thrombolysis systolic BP was significantly associated with poorer 90-day functional outcome (mean difference 3.87 mmHg; 95% CI, 1.18–6.56) and increased incidence of sICH (mean difference 5.31; 95% CI 2.22–8.40) and suggested that more aggressive lowering of BP below the current recommendations before thrombolysis might be beneficial (12). Nevertheless, intensive blood pressure control seemed not safe and feasible.

Blood pressure control in the acute phase has a great benefit on clinical outcomes, with an SBP target of 140–150 mmHg being correlated with favorable outcomes in several previous studies (13, 14). However, the majority of clinical institutions and physicians do not have a standardized protocol for post-stroke BP management (15). The ENCHANTED study (16) identified that intensive blood pressure lowering was safe and led to a reduction in intracranial hemorrhage, however, did not translate into improved clinical outcomes compared with guideline treatment. Nevertheless, the ENCHANTED study did not take recanalization status into consideration. A meta-analysis (17) analyzed pooled data from 13 RCTs, indicating that early blood pressure lowering did not affect the risk of death or dependency at 3 months or the trial endpoint (RR = 1.04; 95%CI: 0.96–1.13; *P* = 0.35), and had a neutral effect on recurrent vascular events, disability or death, all-cause mortality, recurrent stroke, and serious adverse events. Further investigations were warranted to identify the optimal blood pressure post-MT treatment.

Systolic BP of over 185 mmHg is a contraindication to thrombolytic treatment with alteplase in AIS patients, but the causal link between baseline BP in patients treated with IVT still remains uncertain. A *post-hoc* analysis (5) of MR CLEAN observed no interaction between SBP and the effect of intra-arterial treatment on the functional outcome (*P* = 0.90). Forlivesi et al. (18) conducted a retrospective analysis based on data prospectively collected from 200 consecutive anterior ischemic stroke patients treated with IVT. No correlation between clinical outcome post IVT and systolic/diastolic hypertension was observed. Rusanen et al. (19) performed a retrospective analysis with 104 AIS patients with good collateral filling, finding that moderately elevated SBP was associated with good collateral circulation post IVT, however, inversely correlated with 3-month clinical outcome. Yong et al. (20) found an S-shaped relationship between baseline BP with clinical outcome in AIS patients treated with IVT, based on the ECASS trial database. The above studies predated the effect of IVT on patients treated with MT.

### Strengths and limitations

The major strength of this study is the randomized comparison of MT alone vs. MT along with intravenous alteplase in a group of patients that are eligible for both treatments. However, this study also has some limitations. First, this was a *post hoc* analysis, leading to a risk of type I error. Baseline BP was trichotomized into three groups according to previous studies. Second, our study sample consisted exclusively of large vessel occlusion AIS patients, thereafter, may not be generalizable to patients with medium or small vessel occlusion patients. Third, baseline blood pressure was not consecutively monitored which would provide more details. In addition, this trial excluded patients with baseline BP exceeding 185/100 mmHg because of contraindications for intravenous alteplase. Moreover, only Chinese patients were enrolled, with a relatively small sample size compared with the ongoing ENCHANTED-MT study, with over 2,000 cases.

## Conclusions

In successfully revascularized AIS patients attributing with large vessel occlusion, baseline BP is not correlated with intravenous alteplase, nor could it be used for the prediction of functional outcome. Whether intensive blood pressure control benefits still warrant further investigation.

## Data availability statement

The original contributions presented in the study are included in the article/supplementary material, further inquiries can be directed to the corresponding author/s.

## Ethics statement

The studies involving human participants were reviewed and approved by Changhai Ethics Committee. The patients/participants provided their written informed consent to participate in this study.

## Author contributions

ZXi, ZXu, and ZL contributed equally in manuscript drafting and study design and conduction. ZYongw and YP were co-correspondence in study design. LZ, XP, SH, ZYongx, HW, ZYi, DD, ZR, HQ, XY, and LJ contributed in statistical analysis, data collection, and imaging analysis. LS and CA contributed in draft revision, gramma revision, and study design. All authors contributed to the article and approved the submitted version.

## Funding

The DIRECT-MT trial was funded by the Stroke Prevention Project of the National Health Commission of the People's Republic of China and the Wu Jieping Medical Foundation. This subgroup analysis was funded by the Shanghai Sailing Program (20YF1448000).

## Conflict of interest

The authors declare that the research was conducted in the absence of any commercial or financial relationships that could be construed as a potential conflict of interest. The handling editor DC declared a shared affiliation with the authors LS and CA at the time of review.

## Publisher's note

All claims expressed in this article are solely those of the authors and do not necessarily represent those of their affiliated organizations, or those of the publisher, the editors and the reviewers. Any product that may be evaluated in this article, or claim that may be made by its manufacturer, is not guaranteed or endorsed by the publisher.

## Abbreviations

AIS: acute ischemic stroke
ASPECTS: Alberta Stroke Program Early CT Score
BP: blood pressure
DBP: diastolic blood pressure
EVT: endovascular treatment
LVO: large vessel occlusion
ICH: intracranial hemorrhage
IVT: intravenous thrombolysis/alteplase
MAP: mean arterial pressure
mRS: modified Rankin scale
MT: mechanical thrombectomy
SBP: systolic blood pressure.
